# Supplementary material for: Variable temperature processing by plasmodesmata regulates robust bud dormancy release
Source: Nat Commun. 2026 Jan 14;17:348. doi: 10.1038/s41467-025-67260-z (PMC12804978; doi:10.1038/s41467-025-67260-z)
Supplement: Supplementary file 1 — Supplementary Information [file 41467_2025_67260_MOESM1_ESM.pdf]

Supplementary Materials for

Variable Temperature Processing by Plasmodesmata Regulates robust Bud Dormancy release

Shashank K. Pandey[1]†, Tatiana S. Moraes[2]†, Aswin Nair[1]†, Bibek Aryal[1], Abdul Azeez[1,3], Pal Miskolczi[1], Guillaume Maucort[4], Fabrice P. Cordelières[4], Lysiane Brocard[4], Gwendolyn V. Davis[5], Hannah Dromiack[6,7], Swanand Khanapurkar[6,7], Sara I. Walker[6,8], George W. Bassel[5]‡, Emmanuelle M. Bayer[2]‡, Rishikesh P. Bhalerao[1]*

Corresponding author: Rishi.Bhalerao@slu.se

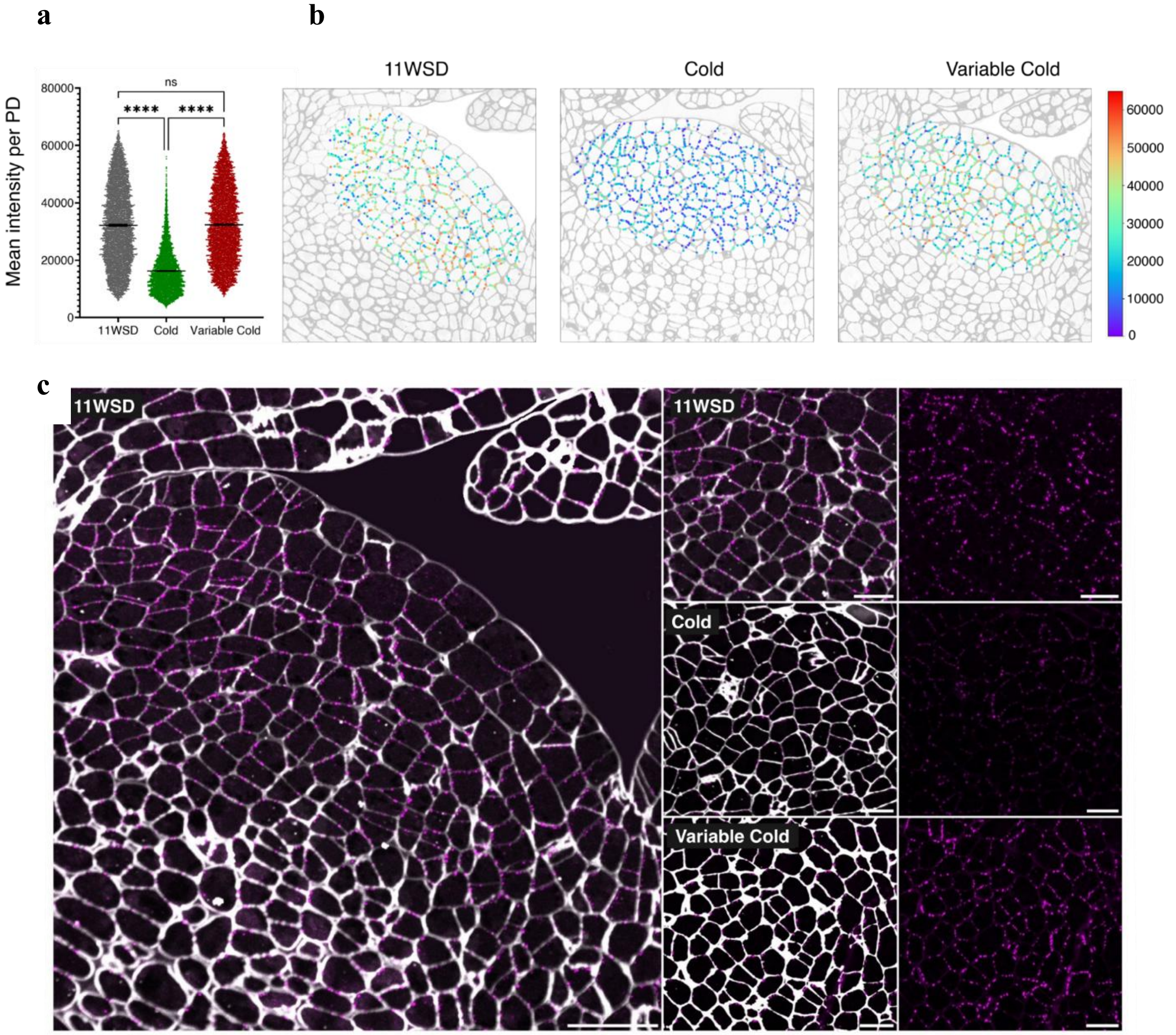

Supplementary Figure 1. PD network responds differentially to constant and variable cold regimes. Quantification and spatial distribution of callose at individual PD, in the shoot apical meristem of aspen buds, after 11 weeks of short days and in response to 4 weeks of constant and variable cold temperatures. **(a)** Graph displaying callose quantification per PD based on mean signal intensity, (data points represent individual PD). Quantification was based on six meristem surfaces per condition, each with an area of 7515 µm², containing 220–280 cells. Statistical analysis was performed using one-way ANOVA ($p < 0.0001$) followed by Tukey's test. Error bars represent the 95% confidence interval of the difference, and asterisks (****) denote a significant difference. **(b)** Visual representation of callose levels at PD. The color legend bar indicates the mean of callose signal intensities. **(c)** Callose immunofluorescence (purple) in semi-thin sections of the aspen shoot apical meristem. Cell walls are shown in grey, stained with the fluorescent dye calcofluor. The purple signal indicates callose accumulation, detected using Alexa Fluor 555-conjugated secondary antibody. Scale bars: 20 µm in the overview image (large square) and 10 µm in the zoomed-in regions (small squares).

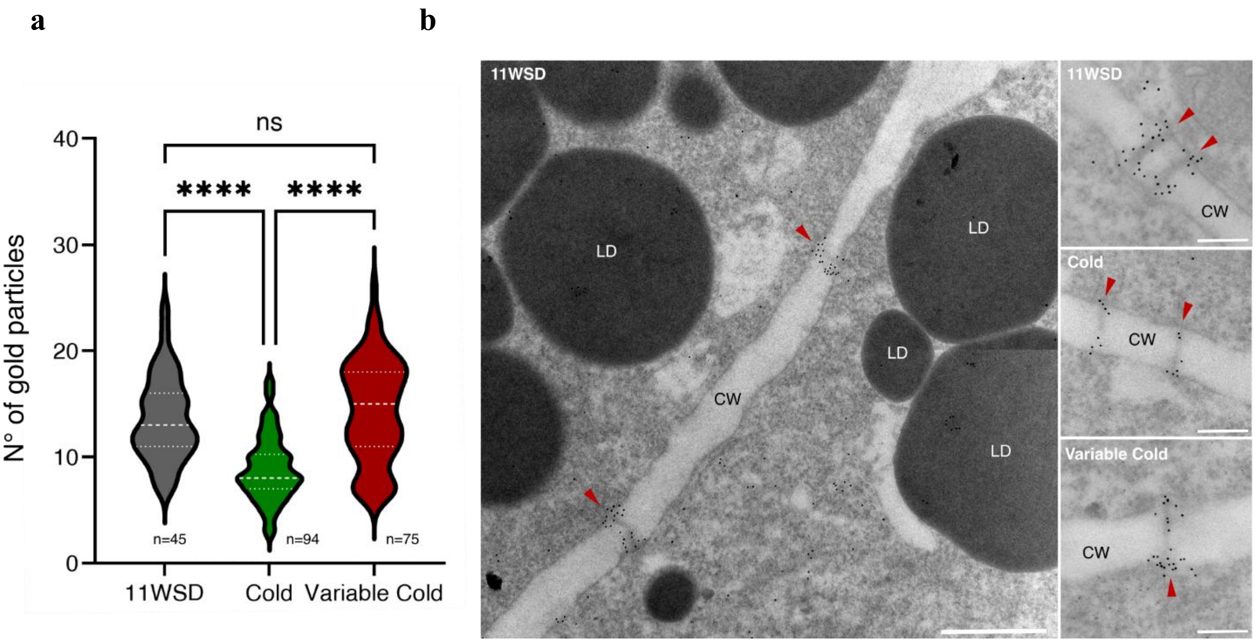

Supplementary Figure 2. Detailed PD structure and callose deposition revealed by immunogold labeling, showing differential responses to constant and variable cold regimes. Samples after 11 weeks of short-day exposure, followed by 4 weeks of either constant or variable cold treatments. **(a)** Callose quantification based on the number of gold particles at individual PD. Statistical analysis was performed using one-way ANOVA ($p < 0.0001$) followed by Tukey's test, asterisks (****) denote a significant difference. Note that 11WSD and Cold values are the same as in Figures 3C. **(b)** High-resolution TEM images with anti-callose labeling (10 nm gold particles). Red arrows indicate PD. CW: cell wall; LD: lipid droplet. Scale bars: 0.4 µm in the overview image (large square) and 0.1 µm in the zoomed-in regions (small squares).

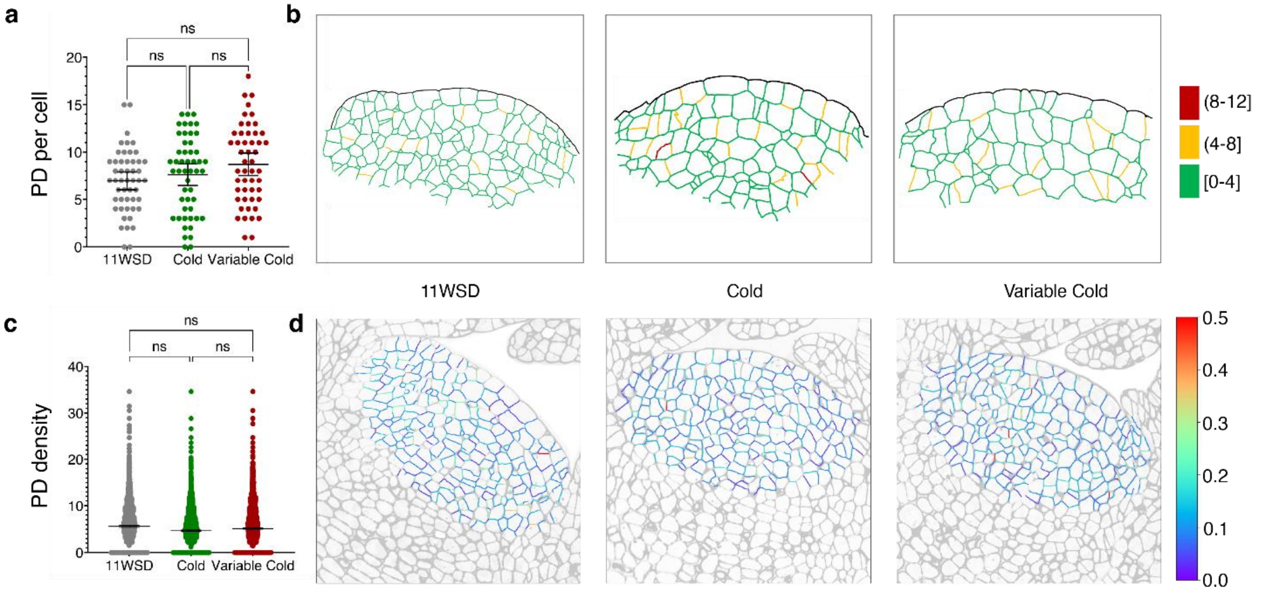

Supplementary Figure 3. PD number and distribution do not change between constant and variable cold regimes. (a-b) PD were imaged by electron microscopy in the shoot apical meristem of aspen buds, after 11 weeks of short days and in response to 4 weeks of constant and variable cold regimes. **(a)** The graph shows the number of PD per cell (n=50 cells). They were not significant based on a one-way ANOVA ($p < 0.05$) and Tukey's multiple comparisons test. Error bars represent the 95% confidence interval of the difference. **(b)** Representative meristem, with the black line representing the outer epidermal wall. Green, yellow, and red lines represent cell-cell interfaces, reflecting the number of PD at each interface, as indicated in the color legend box. **(c-d)** PD were detected by callose immunofluorescence in the shoot apical meristem of aspen buds, after 11 weeks of short days and in response to 4 weeks of constant and variable cold regimes. Each callose dot was extracted as being one PD. **(c)** The graph shows the density of PD per wall. Quantification was based on six meristem surfaces per condition, each with an area of 7515 µm², containing 220–280 cells. Statistical analysis was performed using one-way ANOVA ($p < 0.0001$) followed by Dunnett's test. Error bars represent the 95% confidence interval of the difference. **(d)** Representative meristem, with color-coded cell-cell interfaces. The color legend bar indicates the relative density, normalized to the cell wall length.

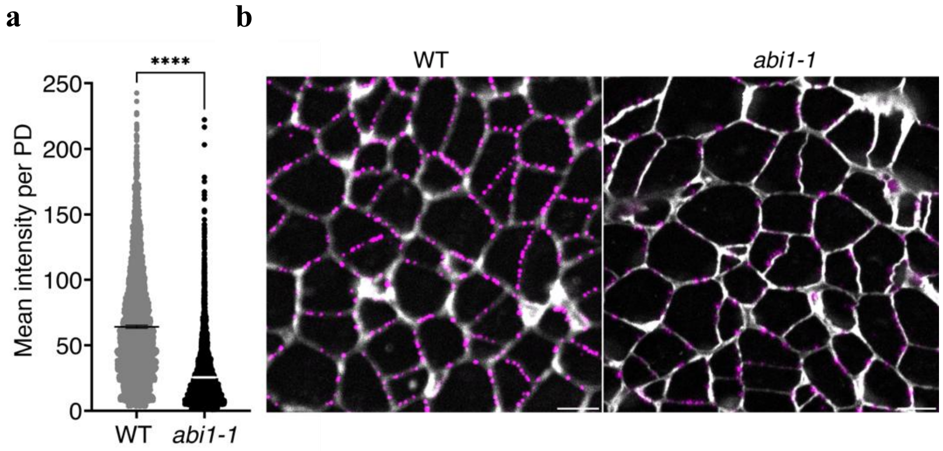

Supplementary Figure 4. Callose quantification at PD in the WT and *abi1-1* shoot apical meristem after 11 weeks of short days. (a) Graph displaying callose quantification per PD based on mean signal intensity, (data points represent individual PD). Quantification was based on six sample surfaces per condition, with analyzed surface areas of 4063 µm². Statistical analysis was performed using Mann-Whitney test ($p < 0.0001$). Error bars represent the 95% confidence interval of the difference, and asterisks (****) denote a significant difference. **(b)** Callose immunofluorescence (purple) in semi-thin sections of aspen tissue visualized by confocal microscopy. Cell walls are shown in grey, stained with the fluorescent dye calcofluor. The purple signal indicates callose accumulation, detected using Alexa Fluor 555-conjugated secondary antibody. Scale bars: 5 µm.

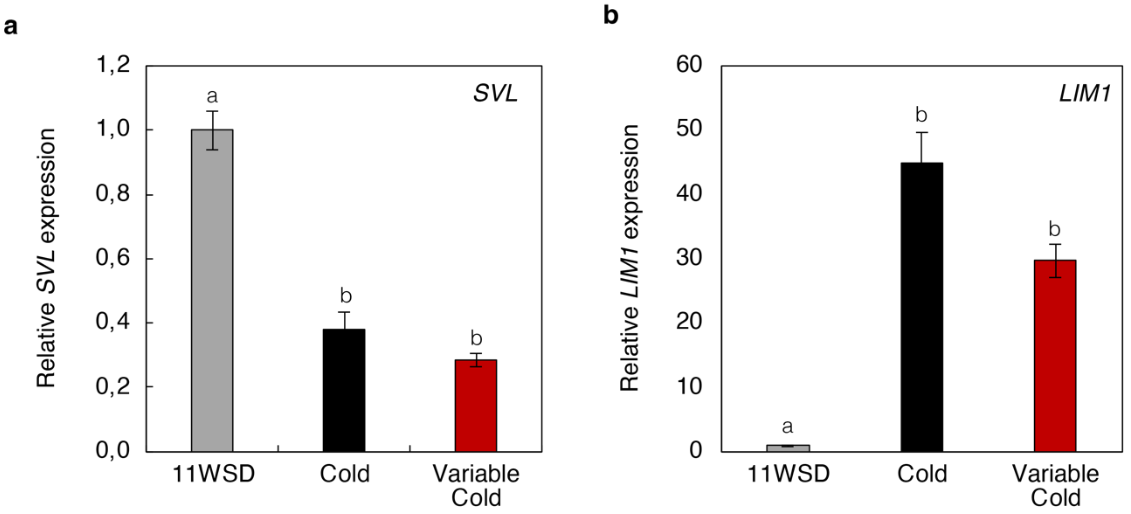

Supplementary Figure 5. *SVL* and *LIM1* expression in constant and variable cold. Relative expression of **(a)** *SVL*, and **(b)** *LIM1* in wild-type buds after 11 weeks of short-day (SD), followed by 4 weeks of constant cold and variable cold temperatures. Expression values shown are normalized to the reference gene UBQ and are averages of three biological replicates. Error bars indicate standard error mean (± SEM). Statistical significance was determined using one-way ANOVA and multiple comparison by the Holm–Sidak method ($P<0.05$). Different letters above the bars indicate significant differences.

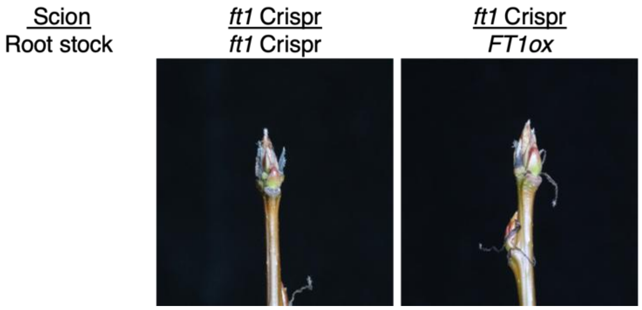

Supplementary Figure 6. Grafting *ft1 Crispr* on FT1ox cannot reactivate growth. *ft1* scions (buds) were grafted onto FT1-expressing stocks (FT1ox) after 5 weeks of cold and moved to LD conditions. The representative pictures of scions were taken after 5 weeks in LD.

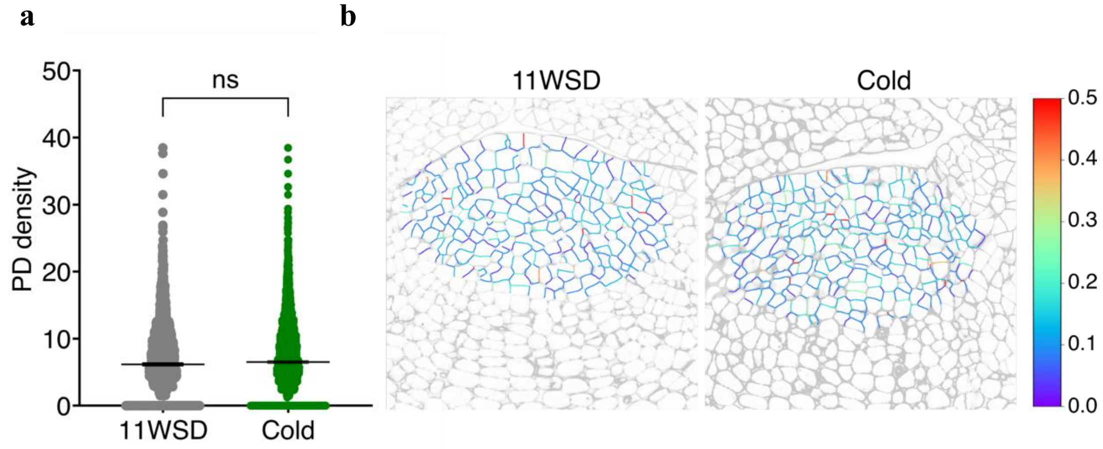

Supplementary Figure 7. PD density do not change during cold treatment in the *ft1* mutant buds. PD were detected by callose immunofluorescence in the shoot apical meristem after 11 weeks of short days and in response to 4 weeks of constant cold regimes. Each callose dot was extracted as being one PD. **(a)** The graph shows the density of PD per wall. Quantification was based on six meristem surfaces per condition, each with an area of 6635 µm². Statistical analysis was performed using Mann-Whitney test ($p < 0.0001$). Error bars represent the 95% confidence interval of the difference. **(b)** Representative meristem, with color-coded cell-cell interfaces. The color legend bar indicates the relative density, normalized to the cell wall length.

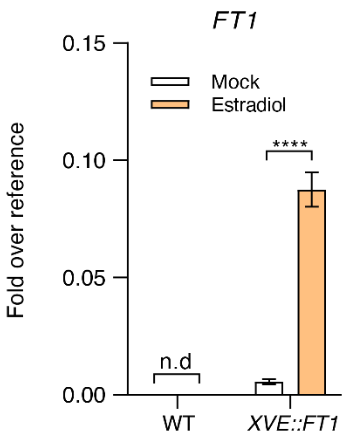

Supplementary Figure 8. *FT1* expression in inducible line. RT-PCR data showing relative levels of *FT1* transcript in the buds of FT1 inducible line *XVE::FT1-GFP-HA* (*XVE::FT1*) treated with mock (DMSO), or 10uM estradiol. All values are means (± SEM) of three biological replicates and are normalized against *UBQ*. Each biological replicate consists of two buds. Plants were shifted to SD conditions to induce bud formation. Estradiol treatment was done in the SD condition and samples were collected after 2 weeks for gene expression. Asterisks indicate significant differences (n.d- not detected, ****p < 0.0001, t-test analysis).

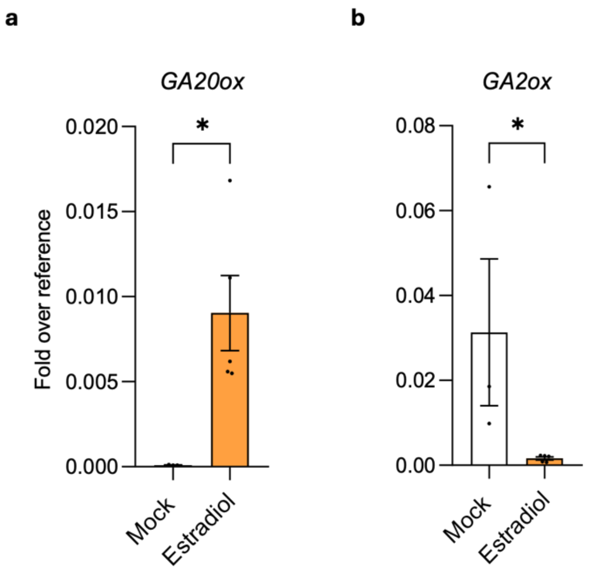

Supplementary Figure 9. FT1 modulates the expression of growth regulator genes. RT-PCR data showing relative levels of **(a)** GA20ox and **(b)** GA2ox transcripts in buds of FT1-inducible lines treated with Mock (DMSO) or Estradiol. Plants were shifted to SD conditions for bud formation, the buds were treated with estradiol in SD and samples were collected after 2 weeks of treatment. Data represents the mean (± SEM) of three or more biological replicates and are normalized to UBQ. Asterisks indicate significant differences (*$p < 0.05$) calculated using t-test.

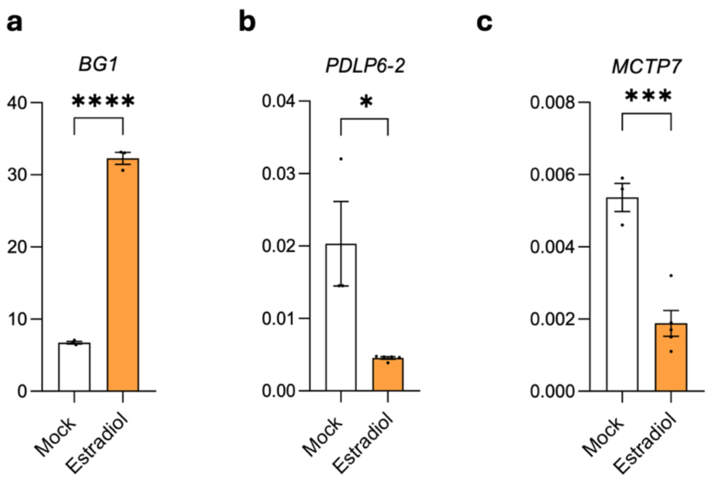

Supplementary Figure 10. FT1 induces the expression of PD regulator genes. (a-c) RT-PCR data showing relative levels of **(a)** β-1,3-GLUCANASE 1, **(b)** PDLP6-2, and **(c)** MCTP7 transcripts in buds of FT1-inducible line (XVE::FT1-GFP) treated with Mock (DMSO) or Estradiol. Plants were shifted to SD conditions for bud formation, the buds were treated with estradiol in SD and samples were collected after 2 weeks of treatment. Data represents the mean (± SEM) of three or more biological replicates and are normalized to UBQ. Asterisks indicate significant differences (*p < 0.05, ***p < 0.001, ****p < 0.0001, ns-not significant) calculated using t-test.

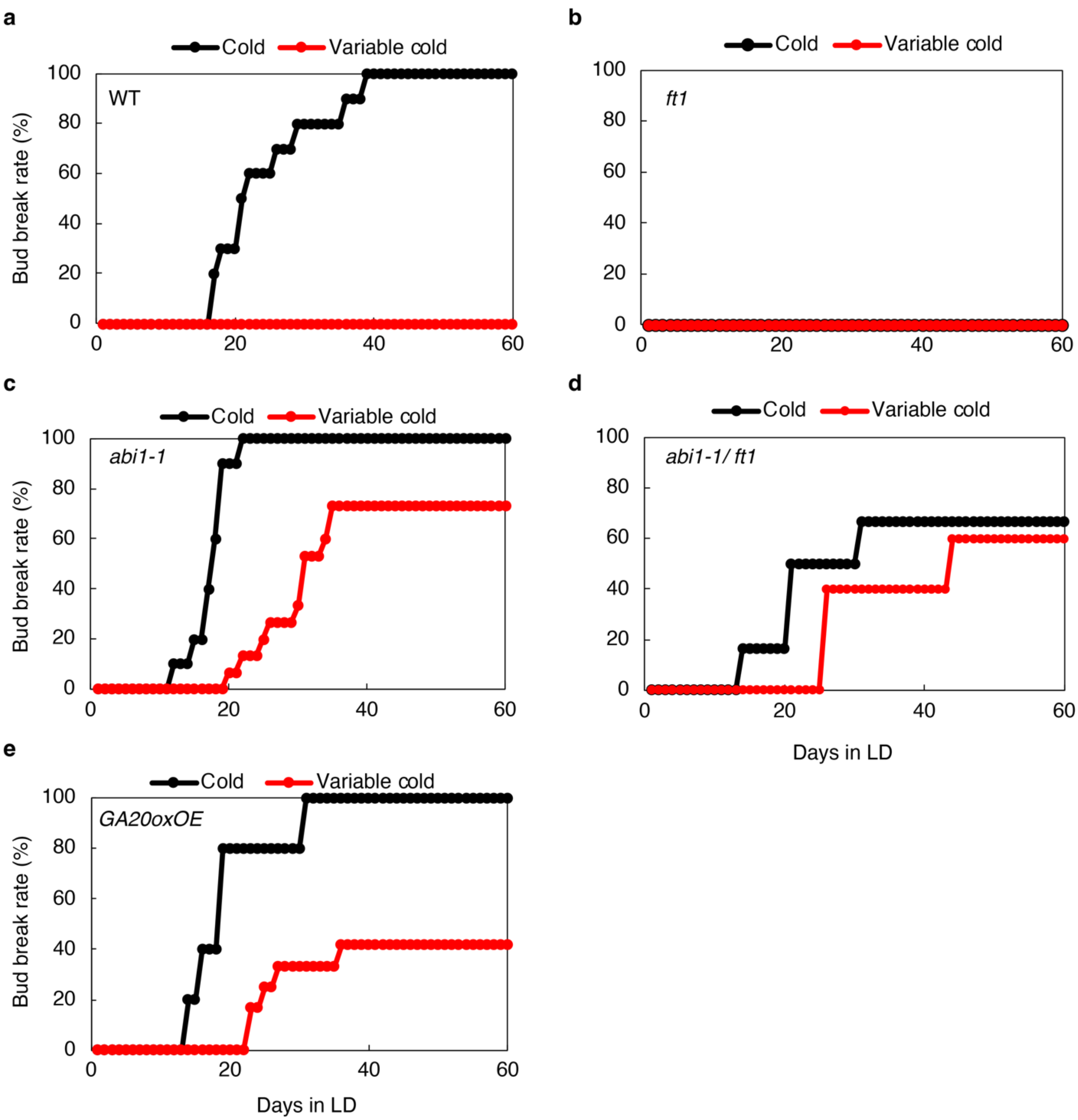

Supplementary Figure 11. Bud break phenotype of *abi1-1*, *ft1*, *abi1-1 /ft1*, and GA20oxOE plants in constant vs. variable cold temperatures. The percent bud break of **(a)** WT, **(b)** *ft1*, **(c)** *abi1-1*, **(d)** *abi1-1/ft1*, and **(e)** *GA20oxOE* plants subjected to constant cold versus variable cold. The experiment was repeated at least twice with similar results, and the percent bud-break is shown with data from 10-15 plants.

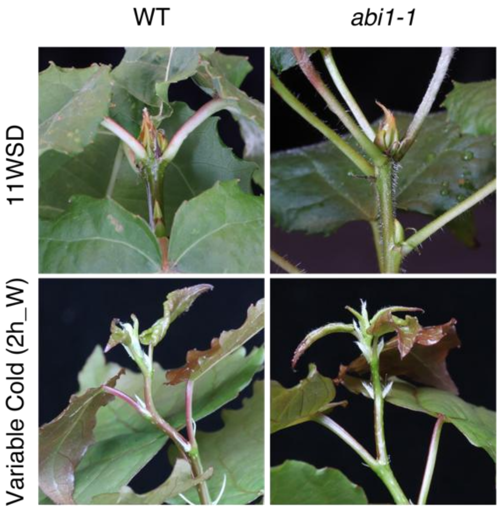

Supplementary Figure 12. Bud break of WT and *abi1-1* plants in constant vs. variable cold (2h_W) temperatures. Plants were grown under short-day (SD) conditions for 11 weeks, followed by exposure to 4 weeks of variable cold (22 hours cold/2 hour warm) and then transferred to warm, long days and bud break was recorded.

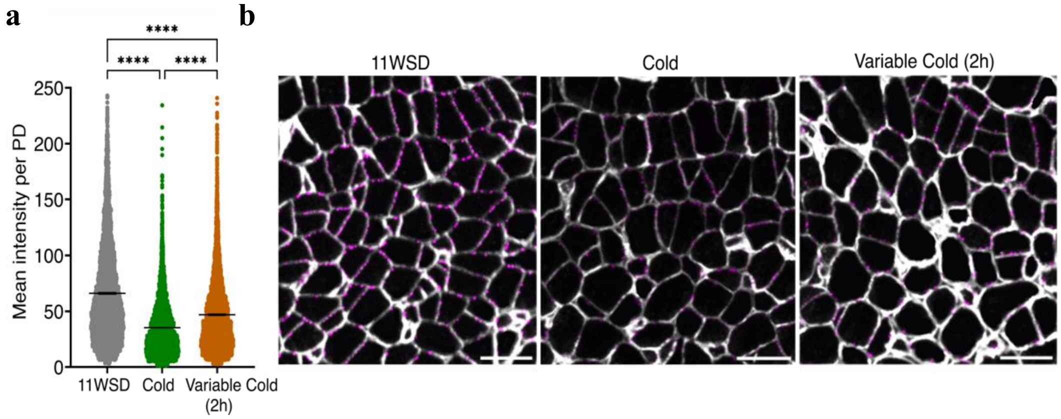

Supplementary Figure 13. Callose quantification at PD in the shoot apical meristem of aspen buds after 11 weeks of short days and in response to 4 weeks of constant or variable cold (22 hour cold at 4°C interrupted by 2 hours at 20 °C daily) temperatures. (a) Graph showing callose quantification per PD based on mean signal intensity (each data point represents an individual PD). Quantification was performed on six sample surfaces per condition, each with an analyzed area of 7515 µm². Statistical analysis was conducted using one-way ANOVA ($p < 0.0001$), followed by Tukey's test. Error bars represent the 95% confidence interval of the difference, and asterisks (****) denote a significant difference. **(b)** Callose immunofluorescence (purple) in semi-thin sections of aspen tissue visualized by confocal microscopy. Cell walls (grey) were stained with the fluorescent dye calcofluor. Callose accumulation (purple signal) was detected using an Alexa Fluor 555 conjugated secondary antibody. Scale bars: 10 µm.

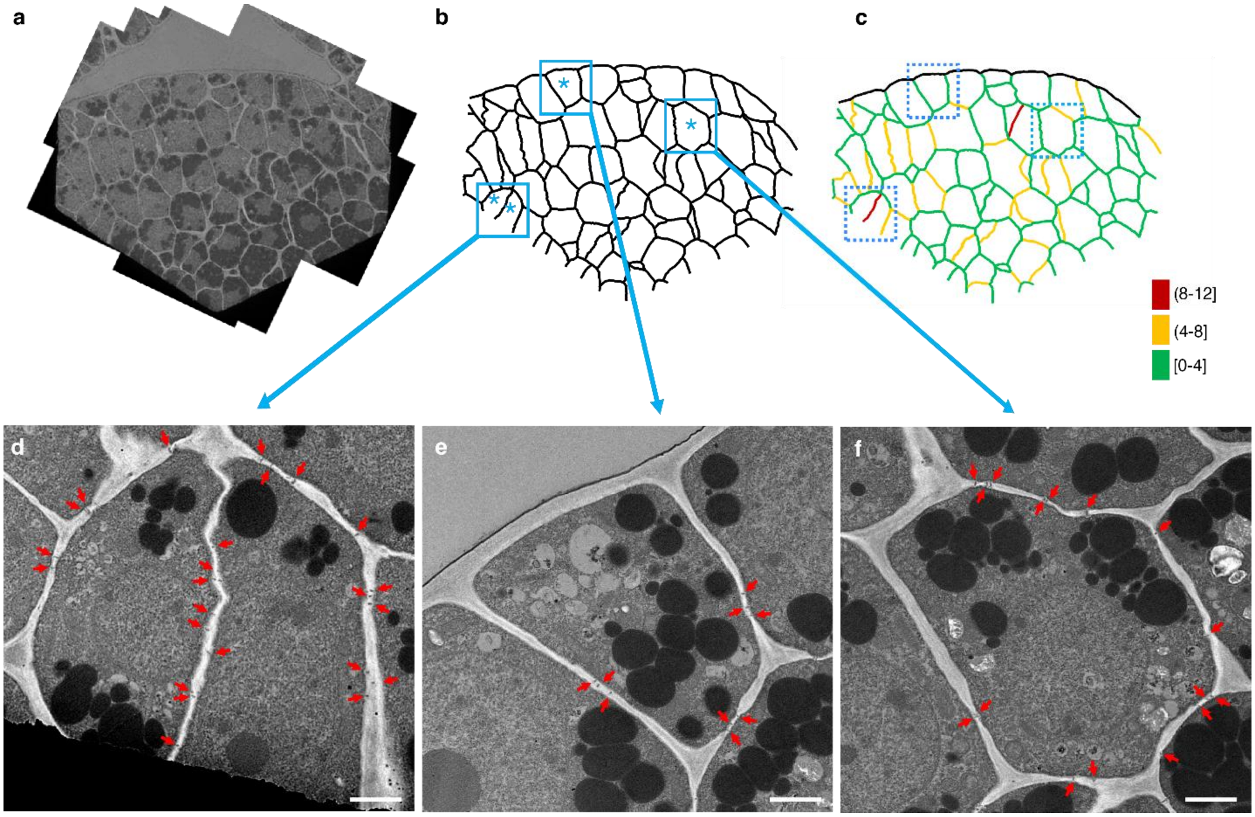

Supplementary Figure 14. Mapping PD distribution and number in the shoot apical meristem of aspen buds using transmission electron microscopy. **(a)** High-resolution individual TEM images were captured and stitched together to reconstruct the entire meristem surface. **(b)** Cell walls were digitally traced and represented as schematic lines. **(c)** Different colors were assigned to the walls based on the number of PD for each section analyzed. The black lines represent the outer epidermal wall. Green, yellow, and red lines indicate cell walls, with colors reflecting the number of PD at cell-to-cell interfaces, as detailed in the color legend boxes. **(d-f)** Close-up views of cells observed by TEM, with red arrows marking PD. Scale bars = 2 µm (D-F).

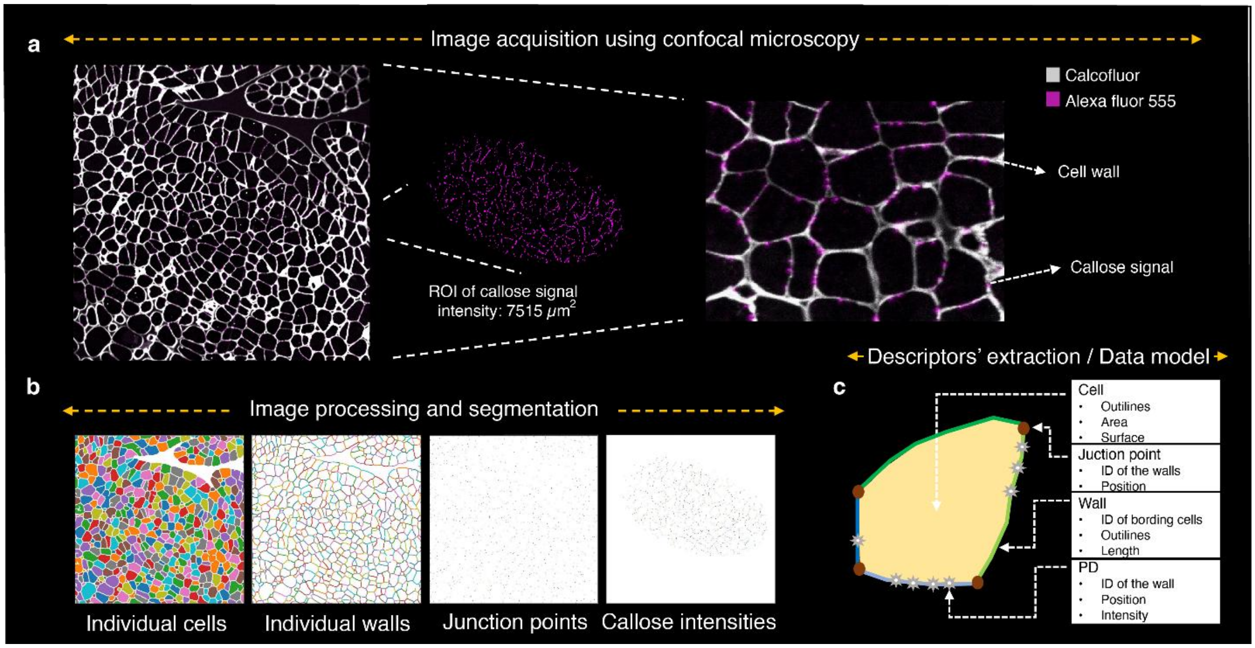

Supplementary Figure 15. Workflow for extracting PD-related data. **(a)** Callose is visualized using callose-immunofluorescence (purple) on semi-thin sections of aspen shoot apical meristem by confocal microscopy. Cell walls, shown in grey, are stained with the fluorescent dye calcofluor. The purple dots represent the callose signal detected via the Alexa fluor 555-conjugated secondary antibody. The callose signal intensity was normalized to an elliptical region of interest measuring 7515 μm². (b) Individual objects in individual images (individual cells, individual walls, junction points, callose dot, and intensity) were pre-processed, identified, and segmented. (c) Descriptors were extracted and stored in a dedicated data container.

Supplementary Table 1. Primers used for qRT-PCR and cloning

Oligos	Sequence (5`- 3`)
PttUBQ-F	GTTGATTTTTGCTGGGAAGCG
PttUBQ-R	GATCTTGGCCTTCACGTTGT
PttGA20ox-F	TTCCACAACGAGAGCGGTCTTG
PttGA20ox-R	TTTGAGAGGCAGGGAAGGGAGAG
PttFT1-F	GCAAGCTTTGGCCATGAAAC
PttFT1-R	GGATATCTTCCTGTTATCGC
TOPO FT1-GFP F	CACCATGTCAAGGGACAGAGATCCTCTGAG
TOPO FT1-GFP R	CTATCCTGCATAGTCCGGGACGTCATAGGGATAGCCCGCATAGTCA<br>GGAACATCGTATGGGTACTTGTACAGCTCGTCCATGC
PttLIM1-F	CTCCAAATGGTCCAAAGGTAC
PttLIM1-R	GCTTCTGTCTTTCTTCCTCTGG
Ptt SVL-F	TGAGAGACTCAAACAGCAAGTGG
Ptt SVL-R	ACTGCCCTTCCTCGTAACCAAC
PttGA2ox-F	CGTGGAGCATAAAGTCGTGG
PttGA2ox-R	GCCCGTTCTTTTGACGTCTT
PttMCTP7-F	TGCTTGCTGTCTGGTATGG
PttMCTP7-R	GTGCGGAGATGATGGAAGAA
PttPDLP6-F	ATGGTGACTGTGCTAGCTGT
PttPDLP6-R	CATCGGCCCACATTTATGCA
PttBG1-F	AGATGATTCCTCCCCTCCCT
PttBG1-R	AGTGACGATGGAGCTGTGAA
